# IL-6 and Neutrophil/Lymphocyte Ratio as Markers of ICU Admittance in SARS-CoV-2 Patients with Diabetes

**DOI:** 10.3390/ijms241914908

**Published:** 2023-10-05

**Authors:** Iulia Făgărășan, Adriana Rusu, Horațiu Comșa, Tudor-Dan Simu, Damiana-Maria Vulturar, Doina-Adina Todea

**Affiliations:** 1 Department of Pneumology, “Iuliu Hațieganu” University of Medicine and Pharmacy, 400332 Cluj-Napoca, Romania; fagarasan_iulia@elearn.umfcluj.ro (I.F.); vulturar.damianamaria@elearn.umfcluj.ro (D.-M.V.); dtodea@umfcluj.ro (D.-A.T.); 2 Department of Diabetes and Nutrition Diseases, “Iuliu Hațieganu” University of Medicine and Pharmacy, 400006 Cluj-Napoca, Romania; 3 Cardiology Department, Clinical Rehabilitation Hospital, “Iuliu Hațieganu” University of Medicine and Pharmacy, 400012 Cluj-Napoca, Romania; dh.comsa@gmail.com; 4 Intensive Care Department, “Leon Daniello” Pulmonology Hospital, 400332 Cluj-Napoca, Romania; tudor.simu@gmail.com

**Keywords:** COVID-19, inflammation, coagulopathy, diabetes mellitus, severity

## Abstract

Inflammation along with coagulation disturbances has an essential role in the evolution towards a severe disease in patients with the coronavirus disease 2019 (COVID-19). This study aimed to evaluate inflammatory and coagulation biomarkers when predicting the need to visit an intensive care unit (ICU) in diabetes mellitus (DM) patients. In a retrospective study, laboratory parameters were examined for 366 participants: ICU = 90, of which 44 patients had DM and no ICU admittance = 276. The ability of inflammatory and coagulation markers to distinguish the severity of COVID-19 was determined using univariate and multivariate regression analysis. In all patients, lactate dehydrogenase was the only predictor for ICU admittance in the multivariate analysis. In the DM group, the results showed that the interleukin (IL)-6 and neutrophil/lymphocyte ratio (NLR) values at admission could predict the need for ICU admittance. Even though there were significant differences between the ICU and no ICU admittance groups regarding the coagulation markers, they could not predict the severity of the disease in DM patients. The present study showed for the first time that the IL-6 and NLR admission values could predict ICU admittance in DM patients. This finding could help clinicians manage the infection more easily if the COVID-19 pandemic strikes again.

## 1. Introduction

The severe acute respiratory syndrome coronavirus-2 (SARS-CoV-2) has been identified as the etiology of an outbreak that occurred in 2020 in Wuhan, China. Although the majority of patients developed mild to moderate symptoms with favorable evolution, a minority of patients with the coronavirus disease 2019 (COVID-19) had severe pneumonia, pulmonary edema, coagulation abnormalities with disseminated intravascular coagulation, acute respiratory distress syndrome (ARDS), septic shock, or even multiple organ failures, requiring hospitalization in the intensive care unit (ICU) or even leading to death [1]. All ages are prone to becoming infected but accumulating evidence has demonstrated that elderly individuals with comorbidities, such as hypertension, diabetes mellitus (DM), and cardio-vascular diseases (CVDs), are especially at a high risk of developing the severe disease, with a poor evolution and prognosis [2,3,4].

DM is a chronic metabolic disease with associated low-grade chronic inflammation [5]. Diabetes itself leads to increased cytokine production, including interleukin (IL)-1, IL-6, IL-8, and tumor necrosis factor-α (TNF-α) [6]. Also, is known to be involved in the dysregulation of the glycosylation of the fragment crystallizable region of immunoglobulin G (IgG Fc) [7]. Given these disturbances in the immune system, patients with DM are more susceptible to viral and bacterial infectious diseases [5,8]. 

During COVID-19, it has been shown that hyperglycemia along with a pre-existing chronic inflammation in DM patients increases the risk of an abnormal immune response and a hyperinflammatory status followed by a cytokine storm [9]. These changes are associated with an increased risk of ICU hospitalization and high mortality [4]. Inflammation has been linked to a prothrombotic status, expressed by a high level of coagulation markers: D-dimer, fibrinogen, and prothrombin time [10]. A high incidence of venous thromboembolism, pulmonary thromboembolism, stroke, or acute coronary syndrome was observed with COVID-19 [11]. In patients with DM, coagulation disorder [12] and endothelial dysfunction are essential risk factors that aggravate the infection. 

Considering that during the SARS-CoV-2 infection, the morbidity and mortality among patients with diabetes were higher compared to the general population (especially for unvaccinated patients), establishing biomarkers that could be used as predictors of severity would be useful from a clinical point of view. Given the broad-spectrum clinical presentation and the potential variability of disease evolution, early recognition of a hyperinflammatory and hypercoagulation state would allow the timely application of preventive measures for a fulminant evolution.

Therefore, this study aimed to evaluate the predictive value of routinely determined inflammatory biomarkers to differentiate severe—with need of ICU—from non-severe cases in patients with DM. Secondary objectives included the evaluation of coagulation markers as predictors of disease severity. 

## 2. Results

### 2.1. Demographic and Baseline Characteristics of ICU Patients and Those Who Did Not Require ICU Admittance

**Figure 1:**
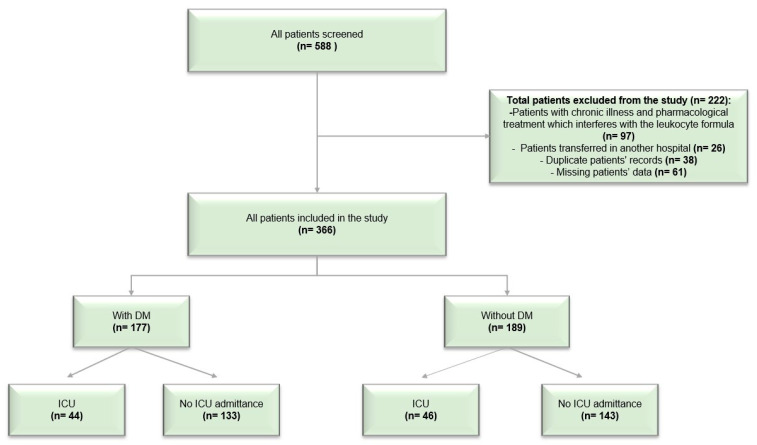
Flow chart of screening and enrolment of the participants.

During this study, 588 patients were hospitalized for the SARS-CoV2 infection. After applying the inclusion and exclusion criteria, 366 were included in this study. Of the total number of participants, 90 were transferred to the ICU during hospitalization (44 with diabetes and 46 without diabetes). Figure 1 presents the flowchart of the participants’ selection criteria and the distribution of the study population. 

The baseline characteristics of patients are summarized in Table 1. The median age was 68.5 (IQR 23-99) years and 228 (62.29%) were men. Of all patients, 177 were known to have type 2 diabetes. Of the total number of participants, 90 patients were admitted to the ICU department (ICU group), of which 44 had diabetes. Patients admitted to the ICU had more frequent obesity (92.22% vs. 82.24%, *p* = 0.001) or advanced-stage abnormalities on chest CT (ground-glass opacities—*p* < 0.001, and total severity score—*p* < 0.001), with a higher rate of mortality in the hospital—62.2% vs. 15.2%, *p* < 0.0001.

The routine blood parameters recorded on the first day of admission were further compared between the ICU and non-ICU admittance groups, as shown in Table 2. Compared to those without ICU admittance, subjects in the ICU group had a significantly higher white blood cell (WBC) count and neutrophilia but lower lymphocyte and platelet counts. Those without ICU admittance had significantly higher lymphocyte levels. Concerning coagulation markers, D-dimers were higher in the ICU group than in the group without ICU admittance, with *p* = 0.001. Thrombocytopenia was more frequently encountered in patients requiring ICU—*p* = 0.049. The platelet-to-albumin ratio (P/Alb) was lower in ICU patients. No significant difference was observed for other tested parameters.

**Figure 2:**
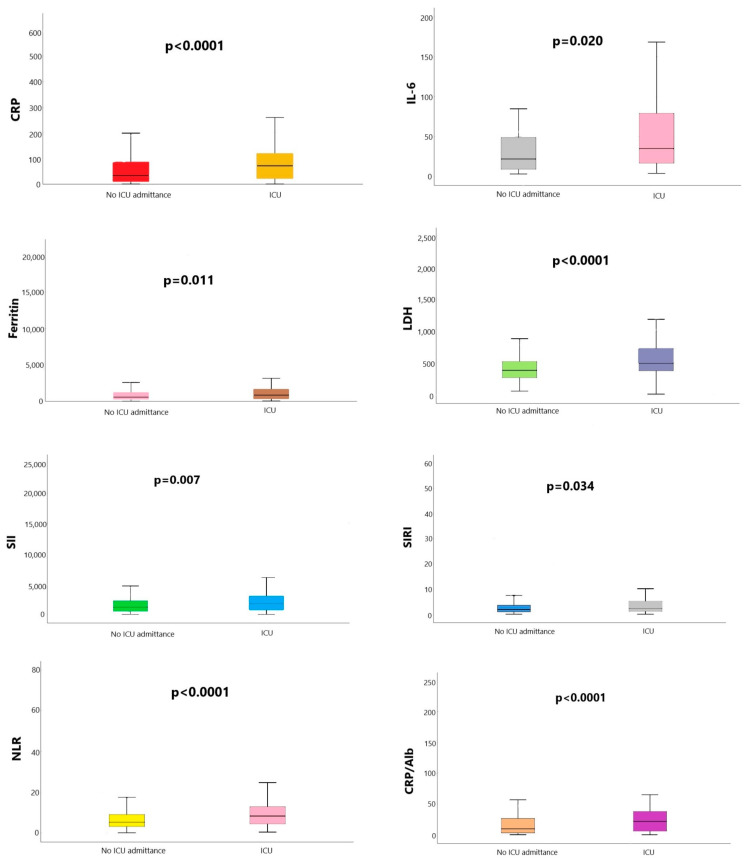
Inflammatory markers between groups. CRP—C-reactive protein; IL6—interleukin-6; LDH—lactate dehydrogenase; SII—systemic inflammation index; SIRI—systemic inflammation response index; NLR—neutrophil/lymphocyte ratio; CRP/Alb—C-reactive protein-to-albumin ratio; ICU—Intensive Care Unit.

The results for inflammatory biomarkers at admittance are presented in Figure 2. The analysis revealed that patients admitted to the ICU department had statistically significant values, with *p* < 0.05, for C-reactive protein (CRP)—71.7 (range; 20.89–120.37) mg/L vs. 33.55 (10.53–86.15) mg/L, IL-6—35.03 (range; 16.9–82.62) pg/mL vs. 21.9 (range; 8.83–48.85) pg/mL, ferritin—842.5 (range; 353.5–1655.5) ng/mL vs. 569.9 (range; 278.9–1203) ng/mL, and lactate dehydrogenase (LDH)—529 (range; 395–755.5) U/L vs. 393 (range; 270.75–550.25) U/L. Also, the ratio value for the systemic inflammation index (SII), systemic inflammation response index (SIRI), neutrophil/lymphocyte ratio (NLR), and CRP to albumin ratio (CRP/Alb) was significantly higher in patients who required ICU admittance, as follows: SII–1830.28 (range; 810.35–3041.31) vs 1214.02 (range; 578.41–2252.46), SIRI—2.35 (range; 1.24–5.18) vs. 1.91 (range; 1.00–7.04), NLR—8.30 (range; 4.51–12.89) vs. 5.27 (3.23–8.98), and CRP/Alb—21.79 (6.18–37.87) vs. 9.77 (3.23–26.59).

### 2.2. Potential Markers for Identifying Severe Cases with Need of ICU Admittance

The biomarkers of interest that were statistically significantly different between the ICU patients and those not requiring ICU admittance were further included in a univariate logistic regression analysis, with the ICU need as a dependent variable. The univariate logistic regression analysis showed that the values of CRP (odds ratio [OR] = 1.006), IL-6 (OR = 1.013), ferritin (OR = 1.000), LDH (OR = 1.002), SII (OR = 1.000), SIRI (OR = 1.053), NLR (OR = 1.074), CRP/Alb (OR = 1.014), and P/Alb (OR = 0.993) were independent predictors of ICU admittance in all patients, whereas D-dimers were not. Subsequently, variables independently associated with ICU admittance in the univariate regression were used as predictors in a multivariate logistic regression model, with ICU admittance as a dependent variable. The multivariate regression model was unadjusted (Model $1^{a}$) and adjusted (Model $2^{b}$) for variables that have been shown to be associated with the severity of the disease: age, gender, body mass index, DM, cardiovascular diseases, hypertension, chronic kidney diseases, respiratory diseases, or vaccination status. The LDH level (OR = 1.002, 95% confidence interval [95%CI]: 1.000–1.005, *p* = 0.044) was the only marker associated with ICU admittance in all participants, independent of the variables correlated with the severity of the infection (Table 3).

### 2.3. Predictors of Severity Correlated with Diabetic Status

#### 2.3.1. Predictors of ICU Admittance in DM Patients

To further investigate the predictors for ICU admittance in the DM subjects, regression analysis was performed for the DM group—Table 4. In the univariate regression, CRP, IL-6, ferritin, LDH, and NLR were significantly associated with ICU admittance, with *p* < 0.05. These were further included in the unadjusted multivariate regression model and NLR was the only variable that remained associated with the increased odds of an ICU admittance. After adjusting for age, gender, body mass index, cardiovascular diseases, hypertension, chronic kidney diseases, respiratory diseases, and vaccination status in the multivariate model, NLR and IL-6 were predictors for ICU admittance (OR 1.228 and 1.028, respectively). 

#### 2.3.2. Predictors for ICU Admittance among Patients without DM

As for patients with DM, we identified predictors of ICU admittance among those without DM using similar variables. The results are presented in Table 5. From the univariate regression analysis, predictors for ICU admittance were associated with increased CRP, D-dimers, SIRI, NLR, and CRP/Alb levels. However, neither in the unadjusted nor in the adjusted multivariate models, the parameters did not remain associated with increased odds for ICU admittance—*p* > 0.05 for all tested variables.

## 3. Discussion

In the present study that evaluated inflammatory and coagulation biomarkers that may qualify as predictors for COVID-19 severity, we showed for the first time that IL-6 can predict severe cases of COVID-19 in patients with diabetes. 

Studies published so far showed that IL-6 is a predictor of severity in COVID-19 patients without diabetes [13,14]. In the severe form of the disease, the immune responses induced by the coronavirus contribute to virus clearance, causing cytokine release syndrome (CRS) [15]. One of the primary inflammatory cytokines is IL-6 [16]. In critically ill patients, it has been shown that high levels of pathogenic T cells and inflammatory monocytes are secreting large amounts of IL-6. These events could trigger an inflammatory storm [17], leading to ARDS [18]. A recent report demonstrated that dehydroepiandrosterone sulfate (DHEAS) has an inhibitory role on IL-6, with a defense immune effect in the SARS-CoV-2 infection [19]. In light of the important role in predicting the severity of COVID-19, it has been proven that patients with diabetes were more likely to receive mechanical ventilation, be admitted to the ICU, and have higher mortality [20]. Moreover, IL-6 contributes to the hypercoagulability status together with TNF-α and IL-1, a phenomenon which, if accompanied by severe inflammatory syndrome, leads to disseminated intravascular coagulation [10,14]. In the SARS-CoV2 infection, there has been an “infection-induced coagulopathy” phenomenon, resulting from hyperactivation of endothelial cells (due to the increased amount of IL-6) and increased release of tissue factor [21]. 

In COVID-19 patients, when the cytokine storm occurs, not only the cytokines rise sharply but other inflammatory markers as well. Hyperinflammation caused by COVID-19 seems to increase NLR levels due to reactive oxygen species released from neutrophils which are causing the cell’s DNA damage [22]. It has been shown that the NLR value is a more sensitive inflammatory marker than the absolute neutrophil and lymphocyte counts [23]. Both neutrophils and lymphocytes are involved in the immune response: inflammation induces neutrophilia, and lymphopenia occurs by suppressing the immune system [24]. In our study, using multivariate regression analysis, we found that NLR could predict the severity of COVID-19 in patients with DM, with results similar to those previously published [25]. The more pronounced increase in NLR in patients with diabetes is due to two mechanisms: the pre-existing chronic inflammation in diabetic patients and the acute inflammation associated with the SARS-CoV-2 infection [26]. A study published by Hussain et al. [27] showed that NLR is associated with higher values for HbA1c, FBG, and CRP in patients with DM. Considering that the COVID-19 infection triggers an important inflammatory syndrome accompanied by increased glycemic values, it can be hypothesized that NLR is also a predictor of glycemic imbalance during hospitalization for patients with diabetes.

In the present study, although the CAR ratio in ICU patients was a predictor for severe disease in the univariate regression, the multivariate regression analysis failed to show a predictive relationship between the severity of infection and CAR. A meta-analysis published by Rathore et al. [28] found that CAR is a predictor of severity in the SARS-CoV-2 infection. The differences may be due to different stages of the inflammatory period in patients analyzed, as Kuluöztürk et al. [29] showed that changes in the levels of acute phase reactants do not appear at the same time in all patients.

We also found significant differences between ICU patients and those without ICU admittance for both SII and SIRI. However, both failed as prognosis markers for the severity of the SARS-CoV2 infection, in line with previous reports [8]. 

The present study also showed that LDH could predict a severe disease in ICU patients, which is similar to the result published by Henry et al. [30]. In line with our findings, Wang et al. [31] reported higher LDH values (*p*-value < 0.001) in ICU compared to non-ICU patients. Considering that in severe/critical SARS-CoV-2 infections some patients developed ARDS, Mesa [32] proved that LDH, alongside thiol and ferritin, is a prognostic biomarker for ARDS development. LDH is an enzyme whose elevated levels indicate the lysis of cells found in different parenchymal organs: heart, liver, muscle, lung, and bone marrow. It was considered a marker of inflammation and a predictor for pneumonia in literature published so far [30]. In severe COVID-19 patients, through inflammatory lesions and cell lysis, increased values are associated with a poor prognosis [30], which is similar to the results presented in this paper. Also, high levels on the first day of admission were correlated previously with new-onset diabetes [33]. Additionally, LDH levels are higher in thrombotic microangiopathy, which is linked in previous studies to renal failure and myocardial injury [34].

Inflammation has a pivotal role in the pathophysiologic mechanism of thrombotic complications in atherosclerosis. In patients with DM, coagulation and endothelial dysfunction are essential factors that aggravate the coronavirus infection [12]. Hypercoagulation, expressed by increased levels of D-dimers, fibrinogen, and abnormalities in prothrombin time (PT), and activated partial thromboplastin time (aPTT), along with thrombocytopenia, are other causes responsible for a poor prognosis, being associated in previous studies with a more severe COVID-19 disease [35,36]. When an imbalance in coagulation pathways occurs, patients with a severe form of disease might develop disseminated intravascular coagulation, with thrombocytopenia as a key element. The hyperinflammatory state observed in COVID-19 destroys bone marrow progenitor cells, with a secondary reduction in platelet production [37]. Another proposed mechanism for thrombocytopenia results from the higher disease severity and degree of lung damage in ICU patients; the impaired lung tissue together with pulmonary endothelial cells could mobilize the lung platelets leading to aggregation and development of microthrombi, with an increase in platelet consumption [37]. High levels of D-dimers were highly correlated with blood clot formation and disseminated intravascular coagulation [36,38]. In recently published literature, a hypercoagulability state expressed by increased D-dimer levels was more frequently associated with mortality in hospitalized patients with COVID-19, as Zhang et al. showed [39]. In the present study, although lower platelet levels and higher D-dimer levels were observed in ICU patients, after adjusting for confounders in multivariate analysis, no association with ICU admittance was observed neither in the DM patients nor in the non-DM group.

This study has several limitations. Firstly, the current paper is a retrospective study, and the data were collected from electronic records; therefore, the accuracy and reliability of the data could vary from subject to subject. Secondly, although the blood laboratory tests were recorded on the first day of hospitalization, subjects could be in different stages of the disease. Thirdly, the small number of DM patients who needed ICU care could provide inaccurate results; so, the present findings should be interpreted with caution. Finally, the findings of this study were described over a considerable period, and variants of the coronavirus could interfere with the results.

## 4. Materials and Methods

### 4.1. Study Design and Participants

The present paper was designed as an observational, analytical, and retrospective study. Data were obtained from the electronic medical record system of “Leon Daniello” Pulmonology University Hospital in Cluj-Napoca, Romania. Consecutive COVID-19 patients (n = 366) admitted to a tertiary Pneumonology University Hospital in Cluj-Napoca, Romania, between 1 April 2021, and 31 January 2022 who met the inclusion criteria and without any exclusion criteria were counted in this study. The inclusion criteria were (1) age > 18 years; (2) a laboratory-confirmed diagnosis of the SARS-CoV2 infection by a real-time-polymerase chain reaction (RT-PCR) of a nasopharyngeal swab; (3) the absence of previously diagnosed chronic illness, which alters the leukocyte formula (e.g., inflammatory chronic disease, autoimmune disease, active cancer, or hematological disorders); and (4) hospitalization > 48 h. Patients excluded from this analysis were those with (1) chronic pharmacological treatment known to affect the leukocyte formula (e.g., chemotherapy or immunosuppressive therapy), (2) duplicate data records, (3) missing clinical, biochemical or radiological findings, or (4) those patients who were transferred to another hospital. 

Data about age, gender, body mass index, and personal medical history of hypertension, diabetes, cardiovascular diseases, respiratory diseases, and laboratory tests were entered into a dedicated electronic database. Results of the following laboratory investigations were collected whenever available: complete blood count, including white blood cell count with leukocyte subtypes, platelet count, cardiac (troponin I, NT pro-BNP), and coagulation markers: D-dimer, fibrinogen, international normalized ratio (INR), activated partial thromboplastin time (aPTT), and prothrombin time (PT); also, inflammatory markers, such as ferritin, CRP, LDH, and outcome during hospitalization: recovery, the need of ICU, intubation, or death. The hemogram-derived ratios were calculated using a part of the complete blood count. While the NLR is calculated by dividing the neutrophil count by the lymphocyte count, the platelet-to-lymphocyte count ratio results from the division of platelets into lymphocytes. A marker that combines the previously mentioned parameters is SII, which is obtained by multiplying neutrophils with platelets and the result is divided by the number of lymphocytes. SIRI is a result of (neutrophils × monocytes)/lymphocytes. The other ratios calculated were fibrinogen divided into albumin, P/Alb, and CRP/Alb.

Also, a CT scan was performed at admission. The CT total severity score was evaluated by lobe involvement for each lung separately, as follows 1-minimal involvement: 1–25%; 2-mild involvement: 26–50%; 3-moderate involvement: 51–75%; severe involvement—76–100% [40]. The decision regarding ICU admission was made according to the Modified National Early Warning Score (Modified NEWS) for COVID-19 patients [41]. To verify the accuracy of patient data collection, two researchers independently double-checked the electronic information.

Participants were divided into two groups: ICU patients and patients without ICU admittance, and each further into DM and non-DM groups. To find the predictors for severe disease in patients with diabetes, in the first phase, we found out the predictors for ICU admittance in the entire population. All statistically significant inflammatory and coagulation markers were subsequently included in the univariate and multivariate analysis for DM and non-DM patients.

### 4.2. Ethics Consideration

This study was designed in accordance with the Declaration of Helsinki and authorized by the Ethics Committee of “Iuliu Hațieganu” University of Medicine and Pharmacy Cluj-Napoca, Romania (approval No 298/29.11.2022). The patient’s consent was not necessary, given the retrospective, non-interventional nature of the study.

### 4.3. Statistical Analysis

Statistical analysis was performed using the IBM SPSS Statistics V26.0 (IBM Corp.: Armonk, NY, USA). The histograms and the Kolmogorov–Smirnov test were used to verify the normal distribution of data. The Student *t*-test and the non-parametric Mann–Whitney U test were used to test the significance of differences in continuous variables between the groups, while the chi-square test and Fisher’s exact test were used for categorical variables. Continuous variables were reported as mean and standard deviation (SD) or as median (25–75% quarters), depending on the normality of the distribution for each variable. Categorical variables were expressed as frequency (percentages).

All parameters with a statistically significant difference between groups were further included in the univariate logistic regression analysis. Variables associated with the need for ICU in univariate analysis were further included in a multivariate logistic regression adjusted for variables that have been shown to be associated with the severity of COVID-19—age, gender, body mass index, cardiovascular diseases, hypertension, chronic kidney diseases, respiratory diseases, and SARS-COV-2 vaccination status. A *p*-value < 0.05 was considered statistically significant.

## 5. Conclusions

Herein we showed for the first time that IL-6 and NLR could predict the severity of the disease in COVID-19 patients with DM. Considering that patients with diabetes present a higher risk of developing a severe form of SARS-CoV2 infection, the present findings emphasize the major importance of identifying patients with an increased inflammatory status from the first day of admission. An early treatment that targets both SARS-CoV-2 infection and antihyperglycemic treatment could reduce the evolution towards a severe form, ketoacidotic coma, and mortality. Therefore, the role of IL-6 in COVID-19 deserves special attention, even if its contribution to predicting the severe case is not fully understood. Further studies are needed to elucidate its role and to determine cutoff values associated with worse outcomes.

## Figures and Tables

**Table 1:** Demographic and radiologic characteristics of the participants.

Characteristics		Total Patients n = 366		ICU n = 90		No ICU Admittance n = 276		
		No.	%	No.	%	No.	%	*p*-Value
Age, years (median; Q1, Q3)		68.5	[23–99]	69	[63–75]	68	[60–77]	0.627
Men, n %		228	62.29	51	56.66	177	64.13	0.205
Comorbidities, n (%)	**Obesity** **(BMI ≥ 30 kg** **/m^2^)**	153	41.8	83	92.22	227	82.24	**0.001**
	Hypertension	133	75.4	70	77.8	206	74.6	0.548
	Cardiovascular disease	24	56.6	54	60	153	55.4	0.448
	Diabetes mellitus	177	48.4	44	48.9	133	48.2	0.908
	Respiratory disease	135	18.6	19	21.1	49	17.8	0.477
**Disease severity**								**<0.0001**
-mild		36	9.8	2	2.2	34	12.3	
-moderate		63	17.2	4	4.4	59	21.4	
-severe		266	72.7	84	93.4	182	65.6	
**Ground-glass opacity (n, %)**		198	54.1	63	70.0	135	48.9	**<0.0001**
**TSS**								**<0.0001**
1		181	49.6	31	34.4	150	54.5	
2		82	22.5	11	12.2	71	25.8	
3		60	16.4	23	25.6	37	13.5	
4		42	11.5	25	27.8	17	6.2	
Vaccinated		34	9.3	7	7.8	27	9.8	0.569
Mechanical ventilation		52	14.2	49	54.4	3	1.1	**<0.0001**
Mortality		98	26.8	56	62.2	42	15.2	**<0.0001**

Data are expressed by median (minimum value–maximum value) or n%. *p* values comparing ICU patients and patients with no ICU admittance; BMI—body mass index; disease severity: mild: clinical symptoms without abnormal radiological findings; moderate: pneumonia on chest computed tomography (CT) without fulfilling any criterion for severe disease; severe: respiratory distress, a respiratory rate ≥30 per minute, SpO_2_ ≤ 93%, or partial pressure of arterial oxygen/concentration of oxygen inhaled (PaO_2_/FiO_2_ ratio) ≤300 mmHg; TSS—total severity score; the sum of acute inflammatory lung lesions involving each lobe was scored as follows: 1—0–25%; 2-mild involvement: 26–50%; 3-moderate involvement: 51–75%; severe involvement—76–100%.

**Table 2:** Laboratory findings at admission.

Parameters	Total Patients n = 366		ICU n = 90		No ICU Admittance n = 276		*p* Value
**White blood cells ×10^3^/L**	8.24	[1.81–39.69]	8.62	[5.39–11.24]	7.06	[5.29–10.01]	**0.023**
**Neutrophil count, ×10^3^/L**	6.87	[0.18–102.3]	6.79	[4.47–9.65]	5.43	[3.73–8.12]	**0.007**
Monocyte count ×10^3^/L	0.42	[0.01–1.37]	0.35	[0.21–0.51]	0.37	[0.26–0.59]	0.084
**Lymphocyte count, ×10^3^/L**	1.35	[0.1–54]	0.84	[0.64–1.15]	1.02	[0.74–1.51]	**0.001**
**Eosinophil count, ×10^3^/L**	0.17	[0–1.98]	0	[0–0.01]	0.005	[0–0.107]	**0.001**
**Platelets count, ×10^3^/L**	245.7	[34.7–634]	205	[159.75–282.25]	230.50	[174.25–308.25]	**0.049**
**D-dimer, µg/mL**	1429.79	[0.08–39698]	807	[434.5–1852.5]	539.5	[321–940]	**0.001**
Fibrinogen, mg/dL	412.65	[317.77–507.95]	415	[314.5–496.25]	412.65	[318.05–513.07]	0.323
Albumin, g/mL	3.30	[3.06–3.69]	3.27	[3.14–3.72]	3.33	[2.96–3.67]	0.262
**Troponin, ng/mL**	0.85	[0.05–5.70]	0.50	[0.05–1.20]	1.03	[0.06–1.30]	**0.011**
NT-proBNP	2148.36	[50–12931]	941	[50–4253]	742	[112.25–3082.75]	0.945
INR	1.06	[0.82–1.65]	1.01	[0.93–1.14]	1.01	[0.91–1.16]	0.613
aPTT (s)	24.02	[17.2–34.9]	27	[22.1–29.2]	22.1	[18.85–25.25]	0.110
Prothrombin time (s)	11.4	[8.1–17.2P]	11.8	[9.3–17.1]	11.3	[8.1–17.2]	0.842
PLR	281.58	[4.82–1754.54]	253.9	[164.05–345.1]	214.96	[143.26–356.78]	0.098
Fbg/Alb	131.17	[48.23–342.58]	120.08	[90.29–149.44]	123.36	[96.51–163.51]	0.265
**P/Alb**	66.66	[50.33–98.05]	60.99	[47.98–85.24]	67.77	[51.81–103.09]	**0.031**

INR—international normalized ratio; aPTT—activated partial thromboplastin time; PLR—platelet/lymphocyte ratio; Fbg/Alb—fibrinogen/albumin ratio; P/Alb—platelet/albumin ratio; s- second.

**Table 3:** Univariable and multivariable logistic regression analysis for detecting the indicators for an ICU admittance in all sample analyzed.

			Model 1 ^*a*^		Model 2 ^*b*^	
Variables	Univariable OR (95%CI)	*p*-Value	Multivariable OR (95% CI)	*p*-Value	Multivariable OR (95% CI)	*p*-Value
CRP	1.006 (1.003–1.009)	<0.0001	1.010 (0.985–1.035)	0.427	0.096 (0.965–1.027)	0.794
IL-6	1.013 (1.002–1.023)	0.017	1.014 (1.000–1.027)	0.044	1.014 (0.999–1.030)	0.070
Ferritin	1.000 (1.000–1.000)	0.006	1.000 (0.999–1.000)	0.348	1.000 (0.999–1.000)	0.328
LDH	1.002 (1.001–1.002)	<0.0001	1.002 (1.000–1.003)	0.083	1.002 (1.000–1.005)	0.044
D-dimer	1.000 (1.000–1.000)	0.091	-	-	-	-
SII	1.000 (1.000–1.000)	0.007	1.000 (1.000–1.001)	0.347	1.000 (0.999–1.001)	0.572
SIRI	1.053 (1.006–1.103)	0.028	0.950 (0.752–1.199)	0.666	1.073 (0.807–1.428)	0.628
NLR	1.074 (1.036–1.112)	<0.0001	1.052 (0.881–1.255)	0.576	1.062 (0.864–1.305)	0.569
CRP/Alb	1.014 (1.004–1.024)	0.008	0.988 (0.921–1.060)	0.735	1.035 (0.945–1.135)	0.457
P/Alb	0.993 (0.986–1.000)	0.044	0.979 (0.951–1.008)	0.161	0.973 (0.939–1.009)	0.136

*a* Model 1: unadjusted for age, gender, body mass index, diabetes mellitus, cardiovascular diseases, hypertension, chronic kidney diseases, respiratory diseases, vaccination status. *b* Model 2: adjusted for age, gender, body mass index, diabetes mellitus, cardiovascular diseases, hypertension, chronic kidney diseases, respiratory diseases, vaccination status. CRP—C-reactive protein; IL-6—interleukin-6; LDH—lactate dehydrogenase; SII—systemic inflammation index; SIRI—systemic inflammation response index; NLR—neutrophil/lymphocyte ratio; CRP/Alb—C-reactive protein-to-albumin ratio; P/Alb—platelet/albumin ratio.

**Table 4:** Univariable and multivariable logistic regression analysis for detecting the indicators for ICU admittance among patients with diabetes.

			Model 1 ^*a*^		Model 2 ^*b*^	
Variables	Univariable OR (95%CI)	*p*-Value	Multivariable OR (95%CI)	*p*-Value	Multivariable OR (95% CI)	*p*-Value
CRP	1.007 (1.001–1.012)	0.014	1.003 (0.996–1.010)	0.376	1.000 (0.989–1.011)	0.976
IL-6	1.022 (1.004–1.041)	0.019	1.016 (0.996–1.036)	0.118	1.028 (1.002–1.055)	0.034
Ferritin	1.000 (1.000–1.001)	0.011	1.000 (0.999–1.001)	0.938	1.000 (0.999–1.001)	0.908
LDH	1.002 (1.001–1.004)	<0.0001	1.002 (0.999–1.004)	0.147	1.003 (0.999–1.006)	0.128
D-dimer	1.000 (1.000–1.000)	0.297	-	-	-	-
SII	1.000 (1.000–1.000)	0.061	-	-	-	-
SIRI	1.026 (0.968–1.088)	0.383	-	-	-	-
NLR	1.070 (1.015–1.128)	0.011	1.120 (1.011–1.241)	0.029	1.228 (1.045–1.443)	0.013
CRP/Alb	1.011 (0.998–1.024)	0.091	-	-	-	-
P/Alb	0.993 (0.984–1.002)	0.134	-	-	-	-

*a* Model 1: unadjusted for age, gender, body mass index, cardiovascular diseases, hypertension, chronic kidney diseases, respiratory diseases, vaccination status. *b* Model 2: adjusted for age, gender, body mass index, cardiovascular diseases, hypertension, chronic kidney diseases, respiratory diseases, vaccination status. CRP—C-reactive protein; IL6—interleukin-6; LDH—lactate dehydrogenase; SII—systemic inflammation index; SIRI—systemic inflammation response index; NLR—neutrophil/lymphocyte ratio; CRP/Alb—C-reactive protein to albumin ratio; P/Alb—platelet/albumin ratio.

**Table 5:** Univariable and Multivariable Logistic Regression Analysis for ICU admittance among patients without diabetes.

			Model 1 ^*a*^		Model 2 ^*b*^	
Variables	Univariable OR (95%CI)	*p*-Value	Multivariable OR (95%CI)	*p*-Value	Multivariable OR (95% CI)	*p*-Value
CRP	1.005 (1.001–1.010)	0.017	1.015 (0.986–1.045)	0.318	1.026 (0.987–1.067)	0.193
IL-6	1.006 (0.994–1.019)	0.323	-	-	-	-
Ferritin	1.000 (1.000–1.000)	0.059	-	-	-	-
LDH	1.001 (1.000–1.002)	0.149	-	-	-	-
D-dimer	1.000 (1.000–1.000)	0.033	1.000 (1.000–1.000)	0.246	1.000 (1.000–1.001)	0.113
SII	1.000 (1.000–1.000)	0.049	1.000 (0.999–1.000)	0.281	1.000 (0.999–1.000)	0.459
SIRI	1.092 (1.012–1.179)	0.024	1.071 (0.908–1.265)	0.414	1.088 (0.889–1.332)	0.412
NLR	1.076 (1.026–1.129)	0.003	1.090 (0.998–1.190)	0.056	1.080 (0.967–1.206)	0.172
CRP/Alb	1.017 (1.002–1.033)	0.028	0.962 (0.869–1.065)	0.457	0.932 (0.812–1.069)	0.315
P/Alb	0.991 (0.980–1.002)	0.123	-	-	-	-

*a* Model 1: unadjusted for age, gender, body mass index, cardiovascular diseases, hypertension, chronic kidney diseases, respiratory diseases, vaccination status. *b* Model 2: adjusted for age, gender, body mass index, cardiovascular diseases, hypertension, chronic kidney diseases, respiratory diseases, vaccination status. CRP—C-reactive protein; IL-6—interleukin-6; LDH—lactate dehydrogenase; SII—systemic inflammation index; SIRI—systemic inflammation response index; NLR—neutrophil/lymphocyte ratio; CRP/Alb—C-reactive protein-to-albumin ratio; P/Alb—platelet/albumin ratio.

## Data Availability

The data presented in this study are available on request from the corresponding author.
